# Real-Time Artificial Intelligence Diagnostic Copilot in Simulated Primary Care Consultations: Randomized Simulation Study

**DOI:** 10.2196/104579

**Published:** 2026-09-22

**Authors:** Ivan Cusacovich, Manuel Pinilla, Ruben Garcia Castro

**Affiliations:** 1 Internal Medicine Department, Hospital Clínico Universitario de Valladolid, Valladolid, Spain; 2 Medsys AI, SL, Madrid, Madrid, Spain; 3 Department of Medical-Surgical Dermatology and Venereology, Hospital Universitario Fundación Jiménez Díaz, Avenida de los Reyes Católicos, 2, Madrid, Madrid, 28040, Spain, 34 915504800

**Keywords:** artificial intelligence, clinical decision support, diagnostic accuracy, automation bias, formative evaluation, simulation study, physician performance, primary care, human-AI interaction, virtual patients

## Abstract

**Background:**

Diagnostic errors in primary care contribute substantially to avoidable morbidity. Real-time, voice-based artificial intelligence (AI) diagnostic assistants may support diagnostic reasoning during clinical encounters, but formative evidence that can be used to assess both diagnostic benefit and overreliance risk before clinical deployment is needed from interactive settings.

**Objective:**

This study aimed to conduct a formative, high-difficulty simulation stress test of whether a real-time, voice-based AI diagnostic assistant was associated with improved physician diagnostic accuracy and measurable overreliance during simulated primary care consultations.

**Methods:**

We conducted a case-level randomized, adjudicator-blinded simulation study in a web-based virtual primary care clinic. Thirteen board-certified family and community medicine physicians managed 260 simulated voice-based consultations. Cases were assigned to either an AI-assisted condition or an unassisted, resource-restricted simulation condition without any external diagnostic aids. No real patients were enrolled, and no clinical care was delivered or modified. The primary outcome was Top-3 diagnostic accuracy, defined as at least one correct diagnosis among up to 3 submitted diagnoses assessed by 3 blinded adjudicators and analyzed using a frequentist binomial generalized linear mixed model fitted by maximum likelihood with random intercepts for physician and case.

**Results:**

All 260 consultations were completed and analyzed. Adjusted Top-3 diagnostic accuracy was 62.3% (95% CI 49.1% to 75.7%) in the unassisted condition and 74.6% (95% CI 63.7% to 85.4%) in the AI-assisted condition (adjusted odds ratio [AOR] 2.68, 95% CI 1.24 to 6.12; χ^2^_1_=6.4, *P*=.01). This corresponds to an adjusted absolute difference of +12.3 percentage points (95% CI +2.7 to +22.6) and a simulation-context number needed to treat equivalent of 8.1 (95% CI 4.4 to 37.2). The post hoc Top-2 analysis was supportive (AOR 2.59, 95% CI 1.23 to 5.77; χ^2^_1_=6.3, *P*=.01). Top-1 point estimates favored AI assistance but were inconclusive: Adjusted accuracy was 47.8% in the unassisted condition versus 57% with AI assistance (AOR 1.83, 95% CI 0.93 to 3.80; χ^2^_1_=3.1, *P*=.08). Leave-one-physician-out and virtual-patient simulator-exclusion sensitivity analyses were consistent with the primary finding. In risk scenarios with an incorrect operative AI suggestion, adjusted concordance was 38.8% in the unassisted condition and 55.8% in the AI-assisted condition, but the between-condition comparison was not statistically significant (AOR 2.08, 95% CI 0.89 to 5.19; χ^2^_1_=2.9, *P*=.09). Mean consultation time increased by 10.7%, and physicians rated the system highly for usefulness and satisfaction.

**Conclusions:**

In this randomized, high-difficulty simulation study, real-time, voice-based AI assistance was associated with higher physician Top-3 diagnostic accuracy than an unassisted, resource-restricted comparator. Some sensitivity analyses were supportive, while others were directionally favorable but inconclusive, and the safety analysis suggested possible overreliance without statistically conclusive difference between conditions. These findings support further refinement and prospective evaluation in real clinical workflows before conclusions are drawn about routine clinical effectiveness or implementation.

## Introduction

### Background

Diagnostic accuracy in primary care is critical, yet missed, delayed, or incorrect diagnoses remain common in ambulatory care and contribute to avoidable morbidity, fragmented follow-up, and increased health care costs [1,2]. Diagnostic difficulty is particularly relevant in primary care, where generalist physicians often evaluate broad, undifferentiated symptom constellations with limited testing at the point of care [3]. Conditions with subtle, nonspecific, or atypical presentations, such as pulmonary embolism or systemic autoimmune disease, are especially vulnerable to delayed or missed diagnosis [4,5]. These challenges create a rationale for tools that support hypothesis generation, information gathering, and diagnostic verification during the consultation.

Large language models (LLMs) have shown strong diagnostic performance in medical question-answering and curated vignette benchmarks, and recent systems increasingly move from single-answer generation toward conversational or workflow-level diagnostic support [6-8]. However, evidence that LLMs improve physician performance is mixed. In a randomized vignette-based study, GPT-4 access did not significantly improve physicians’ diagnostic reasoning compared with conventional resources, despite strong standalone model performance [9]. A later randomized trial found a more modest improvement in patient care tasks when GPT-4 support was embedded in a sequential information environment [10]. More recent evidence suggests that gains may depend on structured AI-literacy training or collaborative diagnostic workflows rather than model access alone [11,12].

Safety evaluation is equally important. LLMs can generate incorrect, unsupported, or biased outputs, and prior studies have documented hallucination, demographic bias, and cognitive bias sensitivity in medical LLM outputs [13-15]. Human-artificial intelligence (AI) interaction studies also show that incorrect AI recommendations can degrade clinician performance even when correct recommendations improve it [16,17]. Interface design is therefore central: Explanations, confidence cues, and recommendation framing may influence trust, but explainability alone does not reliably prevent overreliance [18-20].

Most prior diagnostic support studies have relied on static text vignettes or structured tasks rather than real-time, voice-based consultations. This distinction matters because diagnostic reasoning during a consultation evolves dynamically as physicians ask questions, test hypotheses, and decide what information is relevant. Voice-based assistants and ambient clinical tools are increasingly entering clinical workflows, but much of the existing evidence focuses on documentation, operational efficiency, or constrained clinical tasks rather than diagnostic accuracy and overreliance during an unfolding consultation [21-24]. LLM-powered virtual patients provide a controlled way to evaluate these interactions before exposure to real patients, preserving experimental control while approximating some features of clinical dialogue [25,26].

### Study Objective

Against this background, we conducted a formative, case-level randomized, adjudicator-blinded simulation study to evaluate a real-time, voice-based diagnostic decision-support system during high-difficulty simulated primary care consultations. The study was designed as a diagnostic stress test rather than as an estimate of routine clinical effectiveness. By using interactive virtual-patient consultations, the study aimed to move beyond static vignette testing while preserving a controlled setting in which diagnostic benefit and overreliance risk could be evaluated before prospective clinical deployment.

The primary objective was to assess whether AI assistance to the physician was associated with higher Top-3 diagnostic accuracy than an unassisted simulation condition without external diagnostic aids. A secondary safety objective was to quantify whether physicians were more likely to submit diagnoses concordant with incorrect AI suggestions when those suggestions were visible. These objectives reflect 2 linked questions for formative evaluation: whether real-time AI support can improve diagnostic performance in a challenging simulated setting and whether visible incorrect suggestions create a measurable overreliance signal. Consultation duration, physician-reported usefulness, and overall satisfaction were assessed as formative workflow and acceptability measures; the remaining additional analyses were exploratory or sensitivity analyses.

## Methods

### Study Design

We conducted a formative, case-level randomized, adjudicator-blinded simulation study in a web-based virtual primary care clinic. The study was designed as a high-difficulty diagnostic simulation stress test: Physicians evaluated diagnostically challenging virtual patient cases, and outcomes measured physician diagnostic performance, workflow, acceptability, and overreliance signals rather than patient health outcomes. We hypothesized that real-time, voice-integrated AI assistance would be associated with higher diagnostic accuracy than an unassisted simulation condition without external diagnostic aids, while also increasing the risk that physicians would submit diagnoses concordant with incorrect AI suggestions when those suggestions were visible.

**Figure 1.**
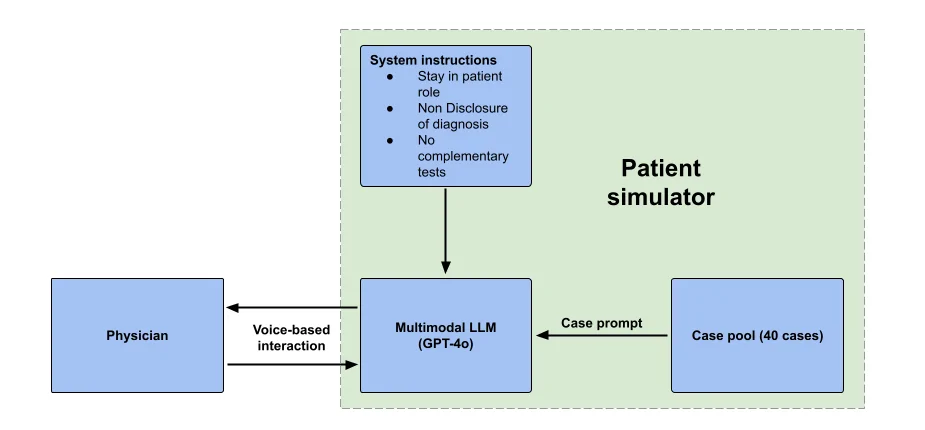
Diagram of the patient simulator’s architecture used for the study. LLM: large language model.

Physicians conducted voice-based consultations with a patient simulator powered by the GPT-4o Realtime API for low-latency voice interaction. Through case-specific prompts, the model was anchored to predefined demographics, symptoms, and permitted primary care physical examination and point-of-care information. The simulator was instructed to behave as a patient rather than as a clinical assistant, to answer only physician-initiated questions, and not to disclose diagnostic labels or unsolicited clinical reasoning. To approximate a constrained primary care visit, it processed requests for predefined primary care point-of-care tools and physical examinations and declined advanced investigations such as imaging or laboratory workups. Pre-study testing was used to check adherence to this constrained role, and a retrospective assessment of that adherence was done based on the transcripts of the conversations. The architecture separated the patient simulator from the AI assistant, ensuring that the AI assistant processed only the live physician-patient dialogue and not the underlying vignette text nor simulator prompts (Figure 1).

For each incoming virtual-patient case, physicians were assigned either to real-time AI support or to the unassisted simulation condition. In the AI-assisted condition, diagnostic support was visible during the consultation; in the unassisted condition, physicians used the same virtual clinic but without AI support or external diagnostic aids. A panel of 3 independent adjudicators, all practicing family physicians, assessed the correctness of diagnoses. A diagnosis was considered correct based on predefined criteria that accepted exact matches, synonyms, or a broader clinical category only when the specific gold standard subtype could not be differentiated with the information available in a primary care setting. Final adjudication was determined by majority rule: When at least 2 of the 3 adjudicators provided the same judgment, their shared judgment was retained as the final classification; otherwise, the assessment was to be discarded.

### Study Registration and Repository

Study registration was not applicable because this was a formative, randomized, simulation-based, physician performance study using virtual patient cases: No real patients were enrolled, no clinical care was delivered or modified, no identifiable patient health data were used, and no patient health outcomes or patient adverse events were measured. All outcomes were physician-level performance, time, usability, or automation-bias measures.

To support transparency and reproducibility, the full study protocol, reporting checklist, simulated clinical vignettes, dataset from all 260 virtual consultations, adjudicator evaluations, statistical analysis code and results, data dictionary, analytical transparency document, virtual patient performance audit, and sensitivity analysis outputs were made publicly available in a permanent repository [27]. The analytical transparency document maps the public statistical code and console outputs to the corresponding manuscript results, enabling end-to-end third-party verification from the locked adjudicator-derived dataset.

### Ethical Considerations

Although the study involved physician participants, it did not involve real patients, the delivery or modification of clinical care, identifiable patient health data, or patient health outcomes and therefore did not fall within the categories requiring ethics committee approval under applicable local regulations. Following completion of the study, the study documentation was reviewed by the Research Ethics Committee of Hospital Universitario La Paz (Madrid, Spain), which issued a formal institutional determination confirming that ethics approval was not required (Reference 57/230428.9/26, dated August 11, 2026). The processing of participating physicians’ personal data complied with Regulation (EU) 2016/679 and Spanish Organic Law 3/2018 [28,29]. Data related to participating physicians were de-identified and stored in an access-restricted Google Drive folder accessible only to the study authors; no directly identifying participant information was included in the adjudication files or the public repository.

All participating physicians provided written informed consent before taking part in the study. Recruitment and consent materials described the evaluated tool, the simulated case format, the expected time commitment, random assignment to AI-assisted and unassisted simulation conditions, and the assessment of diagnostic accuracy. Participation was voluntary, and participants could discontinue participation. Participants received no financial compensation. Access to the evaluated system during the simulation was provided at no cost to participants.

### Simulated Patients and Physician Participants

We selected 40 diagnostically challenging adult clinical vignettes from a peer-reviewed library that was originally validated through supermajority agreement by a panel of 7 independent primary care physicians [30]. This selection was intentional: The case set was used to stress-test diagnostic reasoning under conditions in which missed or delayed diagnoses are plausible, not to reproduce the prevalence or average difficulty of unselected primary care encounters. A physician and 2 LLM-based agents, which were built on a different architecture from the evaluated assistant, selected the cases to reduce content familiarity. The AI assistant was structurally blinded to the original vignette text and underlying simulator prompts, evaluating exclusively the de novo live dialogue dynamically steered by the physician.

Separately, physicians were recruited online through a URL shared with the Madrid regional health service (SERMAS) and social media. Eligibility required active clinical practice in Spain and specialty board certification; computer and internet literacy were implicit eligibility criteria because participation required use of a secure web application and voice interface. Recruitment materials described the evaluated tool, the simulated cases, the expected time commitment, and the AI-assisted and unassisted simulation conditions. A total of 13 board-certified family and community medicine physicians were accepted with no more than one per clinic to reduce cross-contamination. Each physician completed a 30-minute online orientation with the simulation interface before the study.

### Randomization and Masking

Computer-generated randomization occurred at the case level. For each physician, cases were randomly drawn from the 40-case pool and assigned with intended equal probability to either AI assistance or the unassisted simulation condition; no blocking or stratification by physician or case was used, so the achieved distribution could deviate from exact 1:1 balance by chance.

Physicians were necessarily unblinded to AI availability because diagnostic suggestions were visible in AI-assisted consultations and absent in unassisted consultations. The 3 independent adjudicators who assessed diagnostic correctness were blinded: They received only physicians’ diagnoses, AI assistant diagnostic suggestions (also generated in the background for the unassisted condition cases), and the vignette gold standard diagnoses, stripped of all metadata that could identify study arms or indicate whether the AI suggestion had been displayed to the physician. Data analysts worked from the adjudicators’ evaluations and were not blinded; however, they did not influence outcomes, as all statistical modeling was conducted on a locked dataset comprised strictly of the independent adjudicators’ blinded scorings. After blinded adjudication, analytical endpoints were generated through deterministic recoding of the locked adjudicator-derived labels; therefore, the analysts had no ability to alter the primary outcome grading. The statistical code, console outputs, and result-to-manuscript analytical transparency document are available in the public repository [27].

### Procedures

In both conditions, physicians used the same voice interface to conduct simulated consultations. The study was fully web-based and remote: Participants accessed a secure web application from home and were instructed to use a headset in an uninterrupted, quiet environment. To define a controlled, resource-restricted contrast, physicians were instructed not to use nonstudy external diagnostic aids during either condition. The unassisted comparator was intended to isolate the effect of real-time AI assistance under the study conditions and was not intended to represent resource-enabled usual care. After each consultation, physicians submitted between 1 and 3 final diagnoses, which were securely stored. Consultation duration, time stamps, arm assignment, case identifiers, and submitted diagnoses were captured by the web application. After completing their assigned cases, physicians completed an online questionnaire assessing perceived usefulness and overall satisfaction on 5-point response scales. All metadata and arm identifiers were stripped before blinded adjudication. The AI assistant was used under a license provided by the manufacturer.

**Figure 2.**
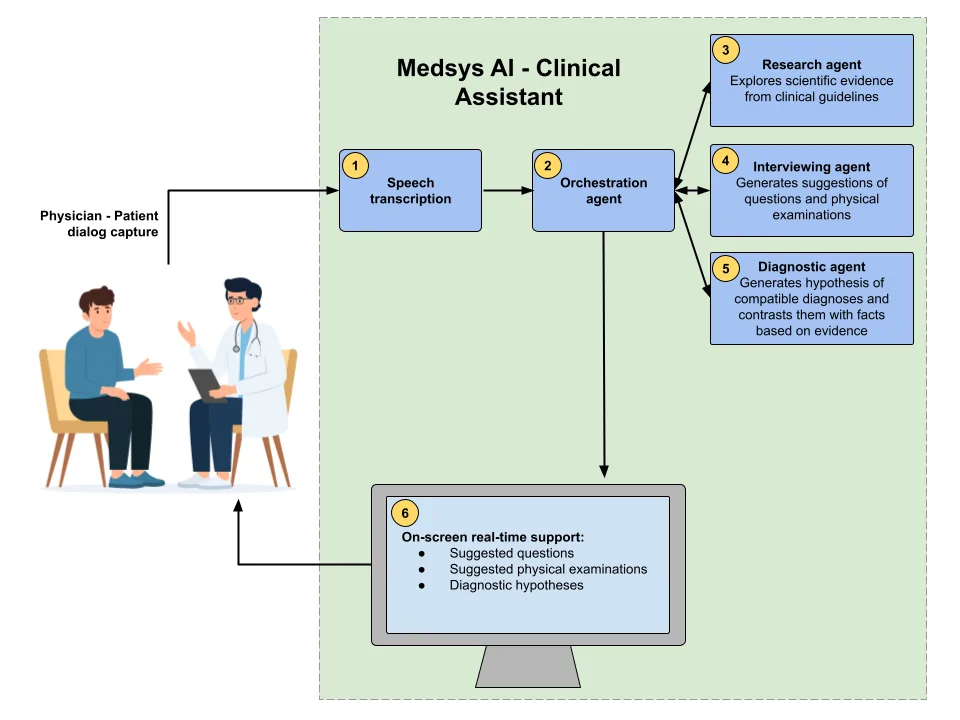
Schematics of the high-level logic architecture and interfaces of the evaluated system. AI: artificial intelligence.

In the AI-assisted condition, physicians received real-time decision support from the Medsys AI-Clinical Assistant V1.0.0 (Medsys AI, SL), the evaluated system, without any functionality change, content update, or bug fix release after study commencement. The system displayed diagnostic suggestions and information-gathering prompts during the consultation while leaving final diagnostic responsibility with the physician (Figure 2). It transcribed the physician-patient conversation and routed it to an orchestration subsystem, which queried a research agent for clinical guideline insights and a diagnostic agent for hypothetical diagnoses. The interface passively displayed a dynamic differential diagnosis with brief rationales, contextually relevant history-taking prompts, and physical examination suggestions. Physicians could use, ignore, or override any displayed content. The system integrates off-the-shelf speech-to-text and generative LLMs, specifically GPT-4o and GPT-4o-mini, which were called concurrently at high frequencies to maintain real-time, low-latency performance. No fine-tuning of these models was performed. Instead, the system relied on retrieval-augmented generation (RAG) anchored to a curated corpus of over 1000 clinical practice guidelines accepted by Spanish medical associations.

### Outcomes

#### Primary Outcome: Top-3 Diagnostic Accuracy

The primary outcome was Top-3 diagnostic accuracy, defined as the proportion of simulated consultations in which the physician provided at least 1 correct diagnosis among their list of up to 3 submitted diagnoses compared with the predefined gold standard for the case. This definition assessed whether the correct diagnosis was present in the physician’s final differential diagnosis rather than whether it was ranked first. Because the simulated consultations ended before access to advanced diagnostic testing, this endpoint was intended to capture whether the correct diagnosis was represented in the end-of-consultation differential that would guide the selection and interpretation of subsequent tests, rather than whether it had already been ranked first. Top-1 and Top-2 accuracy were examined post hoc as complementary measures of diagnostic prioritization.

#### Secondary Safety Outcome: Concordance With Incorrect AI Suggestions

The secondary safety outcome was concordance with incorrect AI suggestions in risk scenarios, defined as consultations in which the AI’s operative diagnostic suggestion was incorrect. Because the AI suggestions were displayed continuously throughout the consultation rather than sequentially, adoption was determined by clinical concordance between the first diagnosis submitted by the physician and the AI’s suggestions classified as high likelihood. This secondary safety outcome compared concordance with incorrect AI suggestions across arms. In the AI-assisted condition, concordance with an incorrect displayed suggestion was interpreted as adoption of that suggestion; in the unassisted simulation condition, concordance with an incorrect background suggestion was interpreted as error concordance rather than true adoption. Because this was a simulated environment, no direct patient-related adverse events were monitored.

#### Formative Workflow and Acceptability Measures

Formative workflow and acceptability measures included consultation duration, which was automatically recorded by the platform, and physician-reported usefulness and overall satisfaction, which were collected after study completion through an online questionnaire using 5-point response scales.

#### Sensitivity and Exploratory Analyses

Sensitivity analyses included post hoc Top-1 and Top-2 diagnostic accuracy, leave-one-physician-out re-estimation, and exclusion of consultations flagged in the virtual patient performance audit. Top-1 diagnostic accuracy was defined as the proportion of consultations in which the physician’s first submitted diagnosis matched the predefined gold standard. Top-2 diagnostic accuracy was defined as the proportion of consultations in which either the physician’s first or second submitted diagnosis matched the predefined gold standard.

Exploratory analyses examined effect modification by case difficulty, physician ability, recent workload, and case position. We used 2 post hoc metrics: case difficulty (defined for each case as one minus the mean unassisted condition accuracy) and physician ability (defined as the physician’s unassisted performance relative to the mean for their specific case mix). Moderation was assessed by testing for statistical interaction between these metrics and the study arm. We also evaluated potential fatigue or familiarization using the case’s sequential position and recent workload and conducted an exploratory comparison of physician performance with standalone AI performance, using background AI outputs generated for all cases.

### Virtual Patient Performance Review

Because simulator behavior was central to the validity of the study, we conducted a retrospective transcript-based audit of virtual patient adherence to its role, assessing diagnostic leakage, excessive helpfulness, fluency or conversational blocking, and adherence to the required physical examination response format [27].

### Statistical Analysis

All hypothesis tests were 2-sided with *α*=.05. All randomized consultations were analyzed according to assigned condition. Case characteristics and case allocation by physician were summarized using counts and percentages. Physician sex was summarized using counts and percentages, whereas physician age and clinical experience were summarized using medians and IQRs. No imputation was required because all randomized consultations had complete outcome data.

#### Primary Outcome: Top-3 Diagnostic Accuracy

The primary endpoint (Top-3 diagnostic accuracy) was analyzed using a frequentist binomial generalized linear mixed model (GLMM) fitted by maximum likelihood with the Laplace approximation. The model included the randomized arm (AI-assisted or unassisted) as a fixed effect, with random intercepts for both physician and case to control for nonindependent observations. From this model, we derived the primary adjusted odds ratio (AOR). Throughout the GLMM analyses, fixed-effect contrasts and interaction terms of interest were evaluated using likelihood-ratio tests against the corresponding nested models. Each comparison differed by 1 fixed-effect parameter and therefore had 1 degree of freedom; the corresponding likelihood-ratio *χ*^2^ statistic is reported alongside each *P* value. The 95% CIs for odds ratios (ORs) were obtained using profile likelihood. We used 30-node Gauss-Hermite integration over the fitted physician and case random-effect distributions to obtain population-averaged marginal probabilities, the absolute risk difference (ARD), risk ratio (RR), simulation-context number needed to treat equivalent (NNT-equivalent), and relative error-rate reduction (RER). To preserve clinical readability, the complete mathematical formulation and execution code are available in our public repository [27]. RER was defined as 1 minus the ratio of error rate in the AI-assisted condition over that in the unassisted condition. Because this was a simulation-based physician performance study, the NNT-equivalent should be interpreted as a contextual measure per simulated consultation, not as an estimate of patient-level clinical benefit. Crude Top-3 diagnostic accuracy was summarized using counts and percentages. Percentile 95% CIs for the marginal probabilities, ARD, RR, and RER were obtained from 10,000 parametric bootstrap replicates that simulated new outcomes and random effects and refitted the complete model. NNT-equivalent CIs were obtained by inversion of the ARD CI and were reported only when the ARD estimate and its entire CI were positive. Interrater agreement for physician- and AI-generated diagnosis adjudication was assessed using the Fleiss κ.

#### Secondary Safety Outcome: Concordance With Incorrect AI Suggestions

The secondary safety analysis was restricted to risk scenarios, defined as consultations in which the AI’s operative diagnostic suggestion was incorrect. Crude arm-specific concordance proportions within risk scenarios were reported with Wilson 95% CIs. A mixed effects logistic regression model estimated the odds of concordance with an incorrect operative AI suggestion, with interpretation differing by condition: adoption of an incorrect displayed suggestion in the AI-assisted condition and error concordance with an incorrect background suggestion in the unassisted simulation condition. Model-adjusted concordance probabilities and the adjusted absolute difference were derived using the same Gauss-Hermite integration and parametric-bootstrap procedure as for the primary outcome; concordance with correct and incorrect operative AI suggestions across all consultations was summarized descriptively by condition.

#### Formative Workflow and Acceptability Measures

Consultation duration was summarized using means and medians with IQRs, and the relative difference in mean duration between conditions was calculated. Differences in mean consultation duration were assessed using Welch *t* tests, with degrees of freedom calculated using the Welch-Satterthwaite approximation. Physician-reported usefulness and satisfaction were summarized descriptively using means and SDs. To assess whether the association between study arm and diagnostic accuracy remained after accounting for consultation duration, the primary mixed effects model was refitted with log-transformed, standardized consultation duration as an additional fixed effect.

#### Sensitivity and Exploratory Analyses

To assess robustness to the achieved physician-level allocation imbalance, we refitted the primary model in leave-one-physician-out sensitivity analyses. For each refitted model, the ARD, RR, and NNT-equivalent were recalculated. The post hoc Top-1 and Top-2 sensitivity analyses used the same mixed effects logistic regression structure as the primary analysis, with Top-1 and Top-2 correctness as binary outcomes. Model-adjusted marginal probabilities and ARDs for Top-1 and Top-2 accuracy were obtained using the same Gauss-Hermite integration and 10,000-replicate parametric-bootstrap procedure as for the primary outcome. In a separate post hoc sensitivity analysis, we refitted the primary model after excluding all consultations with quality performance issues. These issues were identified through a retrospective audit of the patient simulator transcripts. A consultation was flagged when the retrospective audit identified a simulator deviation in any assessed performance domain; all flagged consultations were excluded in this sensitivity refit.

To explore whether the intervention’s effect was moderated by case difficulty or physician ability, we conducted 2 separate analyses. We added an interaction term between the study arm and the post hoc case difficulty or physician ability to the primary mixed effects model. Interaction tests used the continuous case-difficulty and physician-ability metrics. For presentation, consultations were grouped into quartiles of case difficulty, whereas physicians were grouped into quartiles of their unassisted Top-3 accuracy; model-adjusted probabilities and ARDs were estimated within each quartile using the corresponding interaction model, and NNT-equivalents were reported only when the ARD estimate and its entire 95% CI were positive. Because case difficulty and physician ability were derived from unassisted condition performance, these moderation analyses were considered exploratory and hypothesis-generating rather than independent subgroup validations. Recent workload was defined as the number of consultations completed by the same physician during the preceding 2 hours, and case position was defined as the consultation’s ordinal position among the physician’s 20 cases. Both variables were standardized before analysis. Their effects were evaluated in an exploratory mixed effects model including recent workload, case position, study arm, and the corresponding interactions with study arm, using the same physician and case random-intercept structure. The exploratory comparison with standalone AI used a mixed effects logistic regression model including evaluator type (physician vs standalone AI), study arm, and their interaction, with a case random intercept and a physician-specific random effect applied only to physician observations.

Data processing and descriptive analyses were performed in Python 3.11 using pandas 2.2.3, statsmodels 0.14.4, and SciPy 1.15.2. The GLMMs were fitted in R 4.6.1 with lme4 2.0‐6, invoked noninteractively from Python through Rscript.

## Results

### Participant and Case Characteristics

The simulation was conducted between April 1, 2025, and May 20, 2025, during which 13 board-certified family and community medicine physicians from the Madrid regional health service (SERMAS) each evaluated 20 simulated patients through the web-based virtual clinic. Once all simulations were completed, the 3 independent adjudicators evaluated the correctness of the diagnoses.

**Figure 3.**
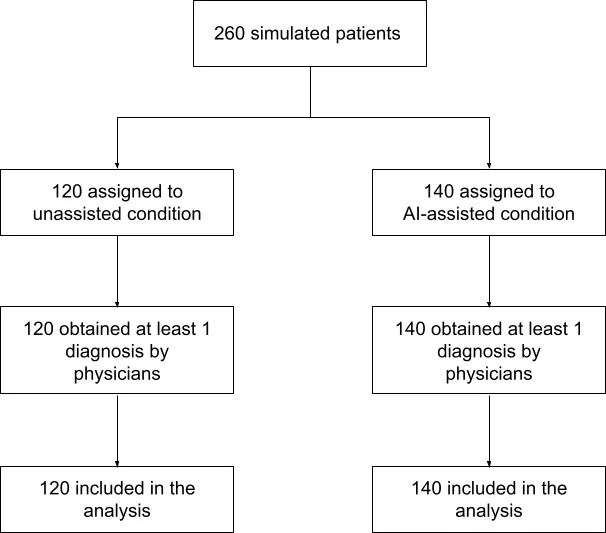
Study flow diagram showing that all 260 randomized consultations were completed and analyzed without any loss reported or detected. AI: artificial intelligence.

Overall, 140 episodes were randomly assigned to the AI-assisted condition, and 120 episodes were randomly assigned to the unassisted simulation condition. This imbalance reflected chance variation from case-level randomization without blocking or stratification. All randomized consultations generated a submitted diagnosis; no consultation required repetition because of a reported technical failure. No episodes were lost or excluded after randomization, and all 260 randomized consultations were adjudicated and analyzed according to assigned condition (Figure 3). The baseline characteristics of the simulated patients are shown in Table 1.

**Table 1.** Baseline characteristics of simulated cases by study condition.

Characteristic	Unassisted condition (n=120), n (%)^a^	AI^b^-assisted condition (n=140), n (%)^a^
Sex		
Women	54 (45)	70 (50)
Men	66 (55)	70 (50)
Age group (years)		
18‐30	28 (23.4)	32 (22.9)
31‐50 years	46 (38.3)	50 (35.7)
>50 years	46 (38.3)	58 (41.4)
Comorbidities		
With comorbidities	67 (55.8)	86 (61.4)
Without comorbidities	53 (44.2)	54 (38.6)
Diagnostic category		
Gastrointestinal	20 (16.7)	18 (12.9)
Endocrinological	16 (13.3)	28 (20)
Neurological and neuromuscular	15 (12.5)	16 (11.4)
Cardiovascular and vascular	14 (11.7)	16 (11.4)
Infectious	13 (10.8)	18 (12.9)
Systemic autoimmune	12 (10)	15 (10.7)
Hematologic and oncologic	9 (7.5)	12 (8.6)
Genetic/respiratory	7 (5.8)	2 (1.4)
Renal	6 (5)	7 (5)
Musculoskeletal	5 (4.2)	2 (1.4)
Obstetric and gynecologic	3 (2.5)	6 (4.3)

a Percentages are column percentages within each category.

b AI: artificial intelligence.

Among the 13 participating physicians, 8 were women (62%) and 5 were men (38%); median age was 30.0 years (IQR 28.0‐43.0). Participants had a median of 6.0 (IQR 4.0‐19.0) years of clinical experience. The distribution of cases among physicians is given in Table 2.

**Table 2.** Distribution of cases per physician and study condition.

Physician	Unassisted condition (n=120), n (%)^a^	AI^b^-assisted condition (n=140), n (%)^a^
1	11 (55)	9 (45)
2	7 (35)	13 (65)
3	7 (35)	13 (65)
4	12 (60)	8 (40)
5	13 (65)	7 (35)
6	5 (25)	15 (75)
7	9 (45)	11 (55)
8	6 (30)	14 (70)
9	9 (45)	11 (55)
10	10 (50)	10 (50)
11	9 (45)	11 (55)
12	12 (60)	8 (40)
13	10 (50)	10 (50)

a Each physician completed 20 consultations. Percentages are row percentages within physician.

b AI: artificial intelligence.

### Outcomes

#### Primary Outcome: Top-3 Diagnostic Accuracy

Diagnoses provided by physicians and those generated by the AI assistance were reviewed and evaluated by 3 independent adjudicators. Interrater reliability among the 3 adjudicators, measured using the Fleiss κ, was κ=0.902 (95% CI 0.864 to 0.937) for diagnoses provided by the physicians and κ=0.896 (95% CI 0.858 to 0.934) for AI-generated diagnoses. The majority rule yielded a final classification for every adjudicated assessment; therefore, no assessment was discarded.

Physicians using AI assistance achieved higher diagnostic accuracy for the primary outcome. A correct diagnosis was provided in 78 of 120 consultations (65%) in the unassisted condition and 105 of 140 consultations (75%) in the AI-assisted condition. In the mixed effects model, adjusted diagnostic accuracy was 62.3% (95% CI 49.1% to 75.7%) in the unassisted condition and 74.6% (95% CI 63.7% to 85.4%) in the AI-assisted condition. Assignment to the AI-assisted condition was associated with higher Top-3 diagnostic accuracy (AOR 2.68, 95% CI 1.24 to 6.12; χ^2^_1_=6.4, *P*=.01), corresponding to an adjusted absolute difference of +12.3 (95% CI +2.7 to +22.6) percentage points, a simulation-context NNT-equivalent of 8.1 (95% CI 4.4 to 37.2), and RER of 32.6% (95% CI 8.9% to 54.4%).

#### Secondary Safety Outcome: Concordance With Incorrect AI Suggestions

For the secondary safety analysis, risk scenarios were defined as consultations in which the AI’s operative diagnostic suggestion was incorrect. In the AI-assisted condition, physicians saw the AI suggestions during the consultation; therefore, a physician diagnostic error concordant with an incorrect displayed suggestion was interpreted as adoption of that suggestion and as an overreliance signal. In the unassisted simulation condition, the AI suggestion was generated only in the background and was not displayed to physicians; therefore, physician error concordant with an incorrect background suggestion was interpreted as error concordance rather than true adoption. In the AI-assisted condition, 51 of 140 consultations were risk scenarios. Physicians submitted a first diagnosis concordant with the incorrect displayed AI suggestion in 29 of these 51 consultations (56.9%, 95% CI 43.3% to 69.5%). In the unassisted simulation condition, 47 of 120 consultations were risk scenarios based on the background AI suggestion, and physicians submitted a first diagnosis concordant with an erroneous AI suggestion in 19 of these 47 consultations (40.4%, 95% CI 27.6% to 54.7%). In the mixed effects model restricted to risk scenarios, the point estimate for concordance was higher in the AI-assisted condition, but the comparison was not statistically significant (AOR 2.08, 95% CI 0.89 to 5.19; χ^2^_1_=2.9, *P*=.09). Adjusted concordance probabilities were 55.8% in the AI-assisted condition and 38.8% in the unassisted condition, corresponding to an adjusted absolute difference of +17.0 percentage points (95% CI –2.7 to +36.8).

When viewed across all consultations, concordance with an incorrect operative AI suggestion occurred in 29 of 140 AI-assisted consultations (20.7%) and in 19 of 120 unassisted consultations (15.8%). Conversely, concordance with a correct operative AI suggestion occurred in 52.1% (73/140) of AI-assisted consultations and 39.2% (47/120) of unassisted consultations. These cohort-wide rates should be interpreted as descriptive measures of concordance with AI outputs; only in the AI-assisted condition can concordance with a displayed incorrect suggestion be interpreted as possible adoption.

#### Formative Workflow and Acceptability Measures

As part of the formative evaluation, we also assessed workflow burden and physician-reported acceptability. Mean consultation time was 328.64 seconds in the unassisted condition and 363.72 seconds in the AI-assisted condition (*t*_257.6_=2.11, *P*=.04), corresponding to a 10.7% increase. Median consultation time was 308.86 (IQR 243.39‐383.17) seconds in the unassisted condition and 341.67 (IQR 278.50‐428.99) seconds in the AI-assisted condition. After adding log-transformed, standardized consultation duration to the primary mixed effects model, the association with study arm remained statistically significant (χ^2^_1_=6.0, *P*=.01), whereas consultation duration was not independently associated with diagnostic accuracy (χ^2^_1_=1.7, *P*=.19).

Physicians rated the evaluated system highly, with a mean score of 4.38 (SD 0.47) for usefulness and 4.46 (SD 0.41) for overall satisfaction on a 5-point scale.

#### Sensitivity and Exploratory Analyses

In the post hoc Top-1 sensitivity analysis, correct Top-1 diagnoses occurred in 61 of 120 unassisted consultations (50.8%) and 79 of 140 AI-assisted consultations (56.4%). Top-1 point estimates favored the AI-assisted condition, but the comparison was not statistically significant (AOR 1.83, 95% CI 0.93 to 3.80; χ^2^_1_=3.1, *P*=.08). Adjusted probabilities were 47.8% in the unassisted condition and 57% in the AI-assisted condition, corresponding to an adjusted absolute difference of +9.2 percentage points (95% CI –1.0 to +20.1). In the corresponding post hoc Top-2 sensitivity analysis, correct Top-2 diagnoses occurred in 74 of 120 unassisted consultations (61.7%) and 100 of 140 AI-assisted consultations (71.4%). The AI-assisted condition was associated with higher Top-2 diagnostic accuracy (AOR 2.59, 95% CI 1.23 to 5.77; χ^2^_1_=6.3, *P*=.01), with adjusted probabilities of 57.9% and 70.7%, respectively, corresponding to an adjusted absolute difference of +12.8 percentage points (95% CI +3.1 to +23.2). Because these analyses were post hoc, Top-2 was interpreted as supportive sensitivity evidence, whereas Top-1 was directionally favorable but inconclusive.

In leave-one-physician-out sensitivity analyses, the direction and magnitude of the primary effect remained stable. Across the 13 refitted models, the adjusted absolute difference ranged from +9.2 to +14.3 percentage points, the adjusted risk ratio ranged from 1.14 to 1.24, and the simulation-context NNT-equivalent ranged from 7.0 to 10.9.

As a result of the retrospective virtual patient performance audit, 16 of 260 consultations were flagged for virtual patient quality deviations related to adherence to role-play instructions. These consultations were excluded in a sensitivity analysis that was done over the remaining 244 consultations. The primary diagnostic effect remained consistent: The AI-assisted condition was associated with higher diagnostic accuracy (AOR 2.78, 95% CI 1.28 to 6.41; χ^2^_1_=6.8, *P*=.009), with adjusted accuracies of 61.4% in the unassisted condition and 74.6% in the AI-assisted condition, corresponding to an adjusted absolute difference of +13.2 percentage points (95% CI +3.3 to +23.6) and a simulation-context NNT-equivalent of 7.6 (95% CI 4.2 to 30.2).

**Figure 4.**
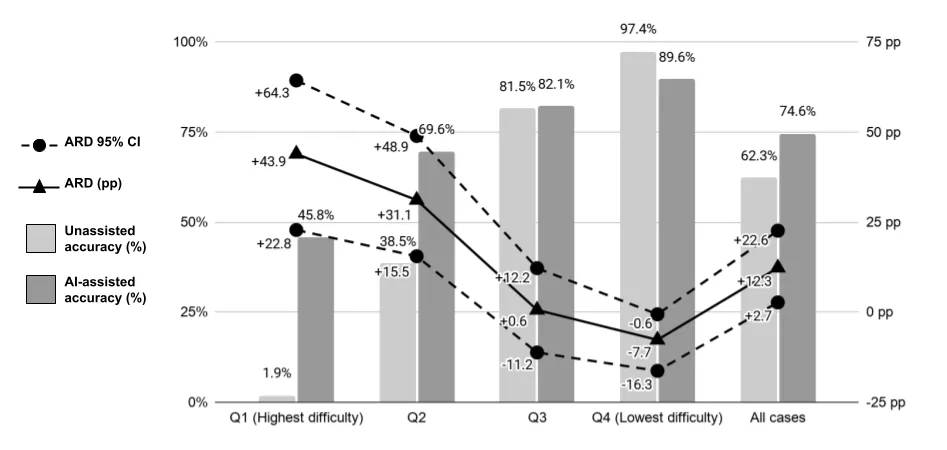
Adjusted diagnostic accuracy and absolute risk difference (ARD) by case difficulty quartile (Q) and overall. Bars show model-adjusted diagnostic accuracy (%) in the unassisted and artificial intelligence (AI)-assisted conditions. Triangles show adjusted absolute risk differences in percentage points, and circles with dashed lines show the corresponding 95% CI bounds.

We computed case difficulty for every case. The weighted mean case difficulty score was similar in the unassisted condition (0.3500, SD 0.3605) and AI-assisted condition (0.3575, SD 0.3716) groups. Two consultations from one case without any unassisted observations were excluded because case difficulty could not be derived for that case. In an exploratory interaction analysis, the association between AI assistance and diagnostic accuracy varied significantly with case difficulty (interaction χ^2^_1_=18.4, *P*<.001). Quartile-specific adjusted accuracies and adjusted absolute differences are shown in Figure 4. The absolute difference was +43.9 percentage points (95% CI +22.8 to +64.3) in the highest-difficulty quartile and +31.1 points (95% CI +15.5 to +48.9) in the quartile 2. No clear difference was observed in quartile 3 (+0.6 points, 95% CI –11.2 to +12.2), whereas the least difficult quartile favored the unassisted condition (–7.7 points, 95% CI –16.3 to –0.6). The simulation-context NNT-equivalent was 2.3 (95% CI 1.6 to 4.4) in the highest-difficulty quartile and 3.2 (95% CI 2.0 to 6.5) in quartile 2; it was not reported for quartiles 3 or 4 because the ARD CI crossed zero or the ARD point estimate was negative.

In a second exploratory analysis, we examined the interaction between study arm and physician ability. The interaction was statistically significant (χ^2^_1_=11.4, *P*<.001), with larger adjusted differences in the lower unassisted-ability strata. The adjusted absolute difference was +34.8 percentage points (95% CI +19.5 to +51.6) in the lowest unassisted accuracy quartile and +10.7 points (95% CI +1.7 to +20.3) in quartile 2; the CIs included no difference in quartile 3 (+2.4 points, 95% CI –7.8 to +12.7) and quartile 4 (–3.0 points, 95% CI –14.6 to +8.4). Because physician ability was derived from unassisted performance and the physician-level variance was estimated at the boundary in this model, these findings were treated as hypothesis-generating.

To assess the influence of workload (the number of cases solved in the preceding 2 h) and consultation order (position among the 20 cases for each physician), we fitted an additional exploratory mixed effects model including standardized recent workload, standardized case position, and their interactions with study arm (Table 3). Point estimates were directionally consistent with a smaller AI effect at higher recent workload and a larger effect later in the sequence, but neither interaction reached statistical significance (Table 3).

To explore how physician performance compared with standalone AI performance, we used a mixed effects logistic regression model including evaluator type (physician vs standalone AI), study arm, and their interaction. In the unassisted condition, the physician versus standalone-AI contrast was not statistically significant (OR 0.56, 95% CI 0.20 to 1.59; χ^2^_1_=1.2, *P*=.27). The human-by-arm interaction was also not statistically significant (OR 2.21, 95% CI 0.68 to 7.35; χ^2^_1_=1.7, *P*=.19), providing no evidence that the physician versus AI contrast differed between conditions. In the AI-assisted condition, physician performance did not differ significantly from standalone AI performance (OR 1.24, 95% CI 0.45 to 3.44; χ^2^_1_=0.2, *P*=.67). These exploratory findings do not establish additive human-AI synergy.

**Table 3.** Exploratory arm-by-workload and arm-by-case-position interaction estimates, with workload and case position standardized prior to analysis.

Interaction	Coefficient	OR (profile-likelihood 95% CI)	Likelihood-ratio χ^2^ (degrees of freedom)^a^	*P* value^a^
Study arm × workload	−0.787	0.46 (0.18‐1.10)	3.0 (1)	.08
Study arm × case position	0.720	2.05 (0.82‐5.40)	2.4 (1)	.12

a *χ*2 statistics and *P* values were obtained using likelihood-ratio tests against the corresponding nested models.

## Discussion

### Principal Findings

In this high-difficulty, simulated primary care setting, real-time access to the AI diagnostic assistant was associated with higher Top-3 diagnostic accuracy than an unassisted, resource-restricted simulation condition. This finding was stable in leave-one-physician-out and virtual patient simulator exclusion analyses; the post hoc Top-2 analysis was supportive, whereas Top-1 was directionally favorable but statistically inconclusive. The study was designed as a diagnostic stress test rather than as an estimate of routine clinical effectiveness; therefore, absolute effect measures, including the simulation-context NNT-equivalent, should be interpreted within the curated challenging case mix.

The study also showed a possible safety-relevant overreliance signal when incorrect AI suggestions were visible, although the adjusted between condition comparison was imprecise and did not reach statistical significance. In the AI-assisted condition, concordance with an incorrect displayed suggestion can reasonably be interpreted as possible adoption of that suggestion. In the unassisted condition, however, concordance with a background AI suggestion reflects error concordance rather than true adoption, because physicians did not see the AI output.

Finally, exploratory analyses suggested marked heterogeneity, with benefit concentrated in more difficult cases and lower unassisted accuracy physician strata, while the easiest case quartile favored the unassisted condition. However, these subgroup findings should be considered hypothesis-generating because they were exploratory and partly derived from unassisted-condition performance.

### Comparison With Prior Work

These findings extend prior studies of LLM-based diagnostic support in 2 ways. First, most randomized evidence has evaluated static text vignettes or structured patient care tasks rather than real-time, voice-based support during an unfolding simulated consultation. In a vignette-based, randomized study, access to GPT-4 did not significantly improve physicians’ diagnostic reasoning compared with conventional resources, even though the standalone model performed well [9]. A later randomized trial of GPT-4 assistance in patient care tasks found a more modest physician-performance improvement in a sequential information environment [10]. Other recent work suggests that stronger benefits may occur when LLM support is paired with AI-literacy training or embedded in collaborative diagnostic workflows [11,12].

Second, this study evaluated an interactive voice-based workflow in which AI suggestions were generated during the consultation while physicians were still gathering information and forming hypotheses. This differs from benchmark studies showing strong standalone LLM performance on curated cases [7,8] and from evaluations focused mainly on passive documentation or operational efficiency. These results therefore support the view that the clinical value of diagnostic AI may depend not only on model capability but also on how model outputs are integrated into physicians’ real-time reasoning workflow.

The overreliance signal is also consistent with prior human-AI interaction research. Studies in digital pathology, radiology, and LLM-assisted diagnostic reasoning have shown that correct AI outputs can improve clinician performance, whereas incorrect outputs can degrade it [16,17,31]. More broadly, automation bias research has long shown that users may overweight automated recommendations in high-stakes environments, especially when systems appear authoritative or are presented as decision-support tools [32,33]. The higher crude concordance with incorrect suggestions in the AI-assisted condition was consistent with a possible overreliance concern in this real-time simulated primary care context, although the adjusted between-condition comparison was not statistically significant.

### Implications for Human-AI Diagnostic Support

The main implication is not that real-time diagnostic AI assistance is ready for routine clinical deployment but that this type of system merits further formative refinement and prospective evaluation. In this controlled simulation, AI assistance appeared to support diagnostic performance. However, point estimates suggested a possible overreliance risk when the displayed suggestion was wrong, although the between-condition comparison was inconclusive. Future systems should therefore be evaluated not only for average diagnostic accuracy but also for how they behave under incorrect-suggestion scenarios and how physicians respond to those errors.

The results also suggest several design priorities. Interfaces should preserve physician verification rather than simply making AI suggestions more salient. Potential mitigation strategies include clearer uncertainty displays, prompts that ask clinicians to consider alternatives before accepting a suggestion, explicit flags when evidence is incomplete, and interface designs that separate hypothesis generation from recommendation endorsement. Training may also be important, because recent randomized evidence suggests that physicians may benefit more from LLM support when they receive AI-literacy training or when collaboration is structured through dedicated workflows [11,12].

Finally, the exploratory comparison with standalone AI should be interpreted cautiously. Neither physician-versus-AI contrast nor the human-by-arm interaction reached statistical significance, so these data do not establish additive human-AI synergy or a difference in relative performance between conditions. Whether specific workflows can produce reliable additive benefit beyond standalone model performance remains an empirical question for future studies.

### Strengths and Limitations

The strengths of this study include case-level randomization, complete inclusion of all randomized consultations, blinded adjudication by 3 independent physicians, excellent interrater reliability, and mixed effects modeling with random intercepts for physician and case. The architecture also separated the patient simulator from the AI assistant, preventing the evaluated assistant from accessing the underlying vignette text or simulator prompts. The use of voice-based virtual consultations represents a more interactive evaluation setting than static text vignettes while retaining the safety and reproducibility advantages of simulation.

The study also has important limitations. First, this was a simulation-based physician performance study, not a clinical trial involving real patients or patient outcomes. Virtual patients cannot fully reproduce pain, anxiety, memory limitations, nonverbal cues, physical examination ambiguity, workflow interruptions, or the consequences of live clinical decisions. The retrospective assessment of the simulated patient performance showed imperfect behavior, which does not invalidate the findings, as demonstrated by the sensitivity analysis, but does underscore the inherently artificial nature of the environment.

Second, the comparator was an unassisted, resource-restricted simulation condition rather than resource-enabled usual care. Although this design standardized the experimental contrast, physicians in routine primary care may consult point-of-care references, clinical guidelines, internet searches, colleagues, or follow-up information. Access to such resources could improve unassisted performance and attenuate the observed relative and absolute contrast. However, the proportion of the observed effect attributable to the resource restriction cannot be estimated from this 2-condition design, because conventional resources could affect both unassisted and AI-assisted performance and may interact with AI use. Accordingly, the reported effect estimates apply specifically to AI assistance versus the resource-restricted comparator and should not be interpreted as the incremental effect of AI over resource-enabled usual care. Future studies should include a conventional resource comparator under the same time constraints and make the same resources available alongside AI assistance. This limitation is especially relevant because the case mix was intentionally difficult. As a result, the observed absolute accuracy gain and simulation-context NNT-equivalent may overestimate the absolute benefit that would be observed in routine care with easier cases and broader diagnostic resources.

Third, the physician sample was modest and recruited from a single Spanish regional health service. Although leave-one-physician-out analyses suggested that the primary result was not driven by a single physician, they address robustness to individual participants rather than external validity. The localized sample limits generalizability to physicians with different training backgrounds, experience, digital familiarity, health care systems, and practice environments. Because participation required use of a web application and voice interface, recruitment may also have favored physicians with greater baseline digital comfort. Future studies should recruit larger physician samples across multiple regions and health care systems and prospectively characterize participants’ digital literacy. The achieved allocation was also imbalanced because randomization was not blocked or stratified by physician or case, although the primary model included physician and case random intercepts and the leave-one-physician-out analyses supported robustness.

Fourth, several analyses were exploratory. Case difficulty and physician ability were derived from unassisted-condition performance, which introduces potential mathematical coupling when those metrics are reintroduced into the models. The workload, case position, physician ability, case difficulty, and standalone AI comparisons should therefore be treated as hypothesis-generating rather than confirmatory. The physician ability and safety models also yielded boundary estimates for the physician-level random effect variance, further supporting cautious interpretation.

Finally, the safety analysis used concordance with incorrect AI suggestions as an operational marker. In the AI-assisted condition, this is a plausible measure of possible overreliance because suggestions were visible. In the unassisted condition, however, the same measure represents background error concordance rather than true adoption. The adjusted 95% CI included no between-condition difference. Accordingly, the between-condition difference should be interpreted as a safety-relevant concordance signal rather than as a clinical harm estimate.

### Conclusions

In this randomized simulation study, real-time, voice-based AI assistance was associated with higher Top-3 diagnostic accuracy than an unassisted, resource-restricted simulation condition. The study also identified a possible overreliance signal when incorrect AI suggestions were visible, although the between-condition safety estimate was imprecise and did not reach statistical significance. These findings support further refinement of diagnostic AI interfaces and prospective evaluation in real clinical workflows before any conclusions are drawn about routine clinical effectiveness or implementation.

## Supplementary material

10.2196/104579 Multimedia Appendix 1 Uptated EHEALTH CONSORT checklist.

## Abbreviations

AI: artificial intelligence
AOR: adjusted odds ratio
ARD: absolute risk difference
GLMM: generalized linear mixed model
LLM: large language model
NNT: number needed to treat
RAG: retrieval-augmented generation
RER: relative error-rate reduction
RR: risk ratio
